# Aqua­bis­(3,5-dimethyl-1*H*-pyrazole-κ*N* ^2^)(oxydiacetato-κ^3^ *O*,*O*′,*O*′′)copper(II) dihydrate

**DOI:** 10.1107/S1600536811015169

**Published:** 2011-05-07

**Authors:** Yan-Li Wang, Guang-Jun Chang, Bing-Xin Liu

**Affiliations:** a Department of Chemistry, Shanghai University, People’s Republic of China

## Abstract

In the title compound, [Cu(C_4_H_4_O_5_)(C_5_H_8_N_2_)_2_(H_2_O)]·2H_2_O, the Cu^II^ cation assumes a distorted octa­hedral coordination geometry formed by two 3,5-dimethyl-1*H*-pyrazole ligands, one oxydiacetate (ODA) dianion and one coordinated water mol­ecule. The tridentate ODA ligand chelates to the Cu cation in a facial configuration with a longer Cu—O bond [2.597 (3) Å], and both chelating rings display envelope conformations. In the mol­ecule, the two pyrazole rings are twisted with respect to each other at a dihedral angle of 57.5 (3)°. Extensive inter­molecular O—H⋯O and N—H⋯O hydrogen bonding is present in the crystal structure.

## Related literature

For background to pyrazole compounds, see: Haanstra *et al.* (1990 ▶); Mukherjee (2000 ▶). For the structure of a related ODA complex, see: Wu *et al.* (2003 ▶).
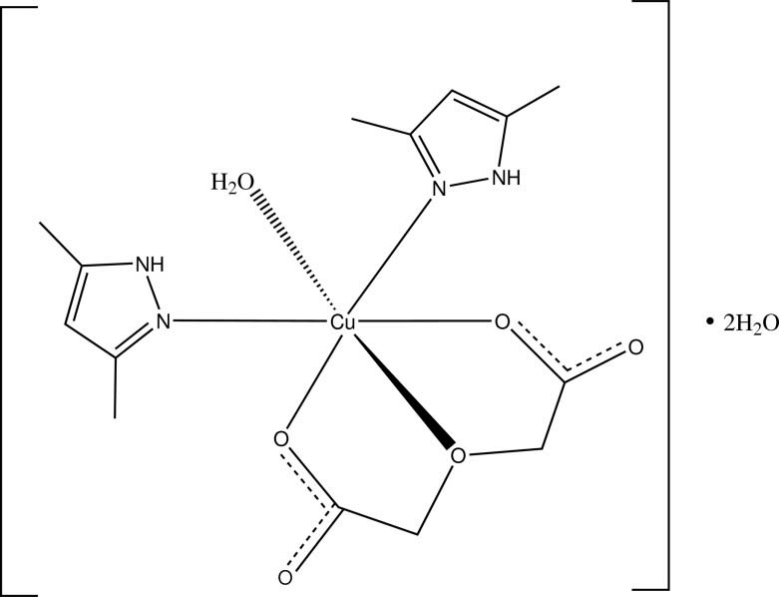


## Experimental

### 

#### Crystal data

[Cu(C_4_H_4_O_5_)(C_5_H_8_N_2_)_2_(H_2_O)]·2H_2_O
*M* *_r_* = 441.93
Triclinic, 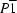

*a* = 7.5502 (12) Å
*b* = 10.6264 (17) Å
*c* = 12.687 (2) Å
α = 92.219 (2)°
β = 104.880 (2)°
γ = 93.769 (2)°
*V* = 980.0 (3) Å^3^
*Z* = 2
Mo *K*α radiation
μ = 1.16 mm^−1^
*T* = 295 K
0.25 × 0.19 × 0.15 mm

#### Data collection

Bruker SMART 1000 diffractometer
Absorption correction: multi-scan (*SADABS*; Bruker, 2001 ▶) *T*_min_ = 0.767, *T*_max_ = 0.840
5085 measured reflections
3389 independent reflections
2663 reflections with *I* > 2σ(*I*)
*R*_int_ = 0.023

#### Refinement

*R*[*F* ^2^ > 2σ(*F* ^2^)] = 0.051
*wR*(*F* ^2^) = 0.133
*S* = 1.05
3389 reflections
244 parameters
H-atom parameters constrained
Δρ_max_ = 0.97 e Å^−3^
Δρ_min_ = −0.64 e Å^−3^

### 

Data collection: *SMART* (Bruker, 2004 ▶); cell refinement: *SAINT* (Bruker, 2004 ▶); data reduction: *SAINT*; program(s) used to solve structure: *SIR92* (Altomare *et al.*, 1993 ▶); program(s) used to refine structure: *SHELXL97* (Sheldrick, 2008 ▶); molecular graphics: *ORTEP-3 for Windows* (Farrugia, 1997 ▶); software used to prepare material for publication: *WinGX* (Farrugia, 1999 ▶).

## Supplementary Material

Crystal structure: contains datablocks I, global. DOI: 10.1107/S1600536811015169/xu5195sup1.cif

Structure factors: contains datablocks I. DOI: 10.1107/S1600536811015169/xu5195Isup2.hkl

Additional supplementary materials:  crystallographic information; 3D view; checkCIF report

## Figures and Tables

**Table 1:** Hydrogen-bond geometry (Å, °)

*D*—H⋯*A*	*D*—H	H⋯*A*	*D*⋯*A*	*D*—H⋯*A*
O1—H1*A*⋯O32^i^	0.85	2.21	2.798 (5)	126
O1—H1*B*⋯O32^ii^	0.85	1.97	2.764 (5)	156
O1*W*—H1*WA*⋯O34	0.85	1.93	2.707 (8)	151
O1*W*—H1*WB*⋯O35^iii^	0.85	2.45	3.097 (8)	133
O2*W*—H2*WA*⋯O32^ii^	0.85	2.23	3.024 (8)	156
O2*W*—H2*WB*⋯O1*W*^iv^	0.88	1.87	2.741 (10)	171
N12—H12*A*⋯O34^iii^	0.77	2.03	2.773 (5)	163
N22—H22*A*⋯O31^ii^	0.75	2.20	2.904 (5)	155

Symmetry codes: (i) 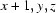; (ii) 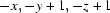; (iii) 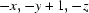; (iv) 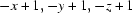.

**Fig. 1.**
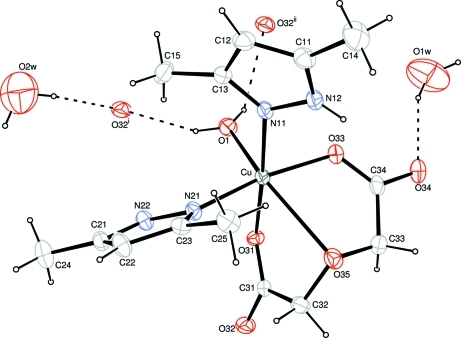
The molecular structure of (I) with 30% probability displacement ellipsoids, dashed lines showing hydrogen bonding [symmetry code: (i) -x, 1-y, 1-z, (ii) 1+x, y, z].

**Fig. 2.**
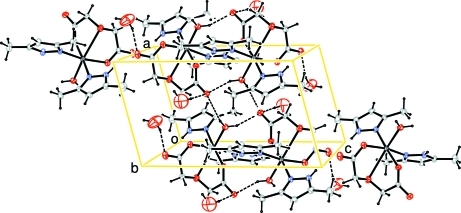
A molecular packing diagram, dashed lines showing the hydrogen bonding between Cu(II) complex molecules.

## supplementary crystallographic information

### Comment

Complexes with pyrazole-based ligands are a frequent subject of chemical investigations giving an opportunity for a better understanding the relationship between the structure and the activity of the active site of metalloproteins (Haanstra *et al.* 1990). Nowadays, attention is paid to the design of various pyrazole ligands with special structural properties to fulfill the specific stereochemical requirements of a particular metal-binding site (Mukherjee, 2000). In our systematic studies on transition metal complexes with the pyrazole derivatives, the title compound was prepared and its X-ray structure is presented here

The molecular structure of the title compound is shown in Fig. 1. The complex has a distorted octahedral coordination geometry formed by two 3,5-dimethyl-1*H*-pyrazole ligands, an oxydiacetate (ODA) dianion and a coordinated water molecule.

Monodentate ligand 3,5-dimethyl-1-H-pyrazole coordinated to the Cu(II) atom by N atoms of pyrazole rings with the 2.015 (4) Å and 1.996 (4) Å of Cu—N bound distance. The adjacent molecules are linked together via O—H···O and N—H···O hydrogen bonding (Table 1) occours between carboxy groups of oxydiacetate dianion and uncoordinated N atom of 3,5-dimethyl-1-H-pyrazole and coordinated water to form the supra-molecular structure as shown in Fig. 2 and Table 1.

The tridentate ODA chelates to Cu(II) atom in a facial configuration, similar to that found in an ODA complex of Cu(II) (Wu *et al.*, 2003). Two carboxyl groups of ODA monodentately coordinate to the Cu(II) atom with the 2.020 (3) Å and 1.959 (3) Å of Cu—O31 and Cu—O33 respectively. Uncoordinated carboxyl oxygen atoms O32 and O34 are hydrogen bonded to the hydrogen atoms of coordinated water of the neighboring complex molecule, as shown in Fig. 2 and Table 1. The uncoordinated carboxyl oxygen atom O32 is hydrogen bonded to the hydrogen atoms of lattice watter molecule and coordinated water of the neighboring complex molecule respectively.

### Experimental

An ethanol-water solution (1:1, 20 ml) containing 3,5-dimethyl-pyrazole-1-carboxamide (0.07 g, 0.5 mmol) and CuCl_2_^.^2H_2_O (0.85g, 0.5 mmol) was mixed with an aqueous solution (10 ml) of oxydiacetic acid (0.07g, 0.5 mmol) and NaOH (0.04g, 1 mmol). The mixture was refluxed for 6 h. After cooling to room temperature the solution was filtered. Blue single crystals of (I) were obtained from the filtrate after 30 d.

### Refinement

Pyrazole H atoms and water H atoms were located in a difference Fourier map and included in the structure factor calculations with fixed positional parameters, and U_iso_(H) = 1.2U_eq_(N) or 1.5U_eq_(O). H atoms on carbon atoms and on oxygen (coordinated and lattice water) were placed in calculated positions, with C—H distances = 0.93 Å (aromatic, pyrazole ring), 0.97 Å (methylene group), 0.96 Å (methyl group), with O—H distances = 0.85 Å, and were included in the final cycles of refinement in riding mode with U_iso_(H) = 1.2U_eq_(C(aromatic and methylene)) and U_iso_(H) = 1.5U_eq_(C(methyl) and O(water)) respectively.

### Crystal data

**Table:**

[Cu(C_4_H_4_O_5_)(C_5_H_8_N_2_)_2_(H_2_O)]·2H_2_O	*Z* = 2
*M**_r_* = 441.93	*F*(000) = 462
Triclinic, *P*1	*D*_x_ = 1.498 Mg m^−^^3^
Hall symbol: -P 1	Mo *K*α radiation, λ = 0.71073 Å
*a* = 7.5502 (12) Å	Cell parameters from 2650 reflections
*b* = 10.6264 (17) Å	θ = 2.0–25.0°
*c* = 12.687 (2) Å	µ = 1.16 mm^−^^1^
α = 92.219 (2)°	*T* = 295 K
β = 104.880 (2)°	Prism, blue
γ = 93.769 (2)°	0.25 × 0.19 × 0.15 mm
*V* = 980.0 (3) Å^3^	

### Data collection

**Table:**

Bruker SMART 1000 diffractometer	3389 independent reflections
Radiation source: fine-focus sealed tube	2663 reflections with *I* > 2σ(*I*)
graphite	*R*_int_ = 0.023
ω scans	θ_max_ = 25.0°, θ_min_ = 2.5°
Absorption correction: multi-scan (*SADABS*; Bruker, 2001)	*h* = −8→8
*T*_min_ = 0.767, *T*_max_ = 0.840	*k* = −10→12
5085 measured reflections	*l* = −15→12

### Refinement

**Table:**

Refinement on *F*^2^	Primary atom site location: structure-invariant direct methods
Least-squares matrix: full	Secondary atom site location: difference Fourier map
*R*[*F*^2^ > 2σ(*F*^2^)] = 0.051	Hydrogen site location: inferred from neighbouring sites
*wR*(*F*^2^) = 0.133	H-atom parameters constrained
*S* = 1.05	*w* = 1/[σ^2^(*F*_o_^2^) + (0.0597*P*)^2^ + 1.5674*P*] where *P* = (*F*_o_^2^ + 2*F*_c_^2^)/3
3389 reflections	(Δ/σ)_max_ < 0.001
244 parameters	Δρ_max_ = 0.97 e Å^−^^3^
0 restraints	Δρ_min_ = −0.64 e Å^−^^3^

### Special details

**Table:**

Geometry. All esds (except the esd in the dihedral angle between two l.s. planes) are estimated using the full covariance matrix. The cell esds are taken into account individually in the estimation of esds in distances, angles and torsion angles; correlations between esds in cell parameters are only used when they are defined by crystal symmetry. An approximate (isotropic) treatment of cell esds is used for estimating esds involving l.s. planes.
Refinement. Refinement of F^2^ against ALL reflections. The weighted R-factor wR and goodness of fit S are based on F^2^, conventional R-factors R are based on F, with F set to zero for negative F^2^. The threshold expression of F^2^ > 2sigma(F^2^) is used only for calculating R-factors(gt) etc. and is not relevant to the choice of reflections for refinement. R-factors based on F^2^ are statistically about twice as large as those based on F, and R- factors based on ALL data will be even larger.

### Fractional atomic coordinates and isotropic or equivalent isotropic displacement parameters (Å^2^)

**Table:**

	*x*	*y*	*z*	*U*_iso_*/*U*_eq_	
Cu	0.03356 (7)	0.40147 (5)	0.29056 (4)	0.02518 (19)	
O1	0.2664 (4)	0.5043 (3)	0.4150 (2)	0.0366 (8)	
H1A	0.3378	0.5675	0.4096	0.055*	
H1B	0.2773	0.4952	0.4826	0.055*	
O31	−0.1202 (4)	0.5234 (3)	0.3466 (2)	0.0273 (7)	
O32	−0.3929 (4)	0.5595 (3)	0.3709 (2)	0.0426 (9)	
O33	0.0910 (4)	0.5157 (3)	0.1841 (2)	0.0346 (7)	
O34	0.0182 (5)	0.6530 (3)	0.0567 (3)	0.0453 (9)	
O35	−0.2706 (4)	0.4198 (3)	0.1414 (2)	0.0373 (8)	
N11	0.1915 (5)	0.2768 (3)	0.2429 (3)	0.0283 (8)	
N12	0.1713 (5)	0.2466 (4)	0.1347 (3)	0.0334 (9)	
H12A	0.1038	0.2781	0.0888	0.040*	
N21	−0.0679 (5)	0.2709 (3)	0.3726 (3)	0.0291 (8)	
N22	−0.0561 (5)	0.2859 (3)	0.4817 (3)	0.0305 (9)	
H22A	−0.0139	0.3484	0.5105	0.037*	
C11	0.2563 (7)	0.1441 (4)	0.1202 (4)	0.0381 (12)	
C12	0.3380 (7)	0.1053 (5)	0.2215 (4)	0.0395 (12)	
H12	0.4079	0.0362	0.2375	0.047*	
C13	0.2950 (6)	0.1905 (4)	0.2959 (4)	0.0324 (10)	
C14	0.2536 (9)	0.0916 (6)	0.0080 (4)	0.0608 (17)	
H14A	0.1822	0.1420	−0.0459	0.091*	
H14B	0.1998	0.0062	−0.0022	0.091*	
H14C	0.3770	0.0932	0.0005	0.091*	
C15	0.3537 (8)	0.1923 (5)	0.4175 (4)	0.0482 (14)	
H15A	0.3035	0.2616	0.4476	0.072*	
H15B	0.4854	0.2020	0.4417	0.072*	
H15C	0.3099	0.1144	0.4414	0.072*	
C21	−0.1237 (7)	0.1816 (4)	0.5189 (4)	0.0326 (10)	
C22	−0.1789 (7)	0.0966 (4)	0.4317 (4)	0.0379 (12)	
H22	−0.2307	0.0148	0.4321	0.046*	
C23	−0.1438 (6)	0.1538 (4)	0.3425 (4)	0.0299 (10)	
C24	−0.1240 (9)	0.1774 (6)	0.6367 (4)	0.0567 (16)	
H24A	−0.0728	0.2569	0.6743	0.085*	
H24B	−0.0514	0.1112	0.6692	0.085*	
H24C	−0.2478	0.1614	0.6422	0.085*	
C25	−0.1799 (7)	0.1017 (5)	0.2272 (4)	0.0418 (12)	
H25A	−0.1402	0.1643	0.1838	0.063*	
H25B	−0.3091	0.0794	0.1984	0.063*	
H25C	−0.1135	0.0280	0.2253	0.063*	
C31	−0.2937 (6)	0.5131 (4)	0.3171 (3)	0.0293 (10)	
C32	−0.3894 (6)	0.4403 (5)	0.2100 (4)	0.0374 (11)	
H32A	−0.4406	0.3592	0.2257	0.045*	
H32B	−0.4904	0.4865	0.1712	0.045*	
C33	−0.2287 (7)	0.5301 (5)	0.0895 (4)	0.0425 (13)	
H33A	−0.2890	0.5996	0.1134	0.051*	
H33B	−0.2789	0.5156	0.0112	0.051*	
C34	−0.0253 (7)	0.5683 (4)	0.1126 (3)	0.0328 (11)	
O1W	0.3682 (9)	0.7458 (7)	0.0748 (6)	0.147 (3)	
H1WA	0.2788	0.6940	0.0771	0.221*	
H1WB	0.3837	0.7389	0.0109	0.221*	
O2W	0.4093 (11)	0.1824 (6)	0.7222 (6)	0.155 (3)	
H2WA	0.3974	0.2410	0.6778	0.232*	
H2WB	0.4868	0.2111	0.7836	0.232*	

### Atomic displacement parameters (Å^2^)

**Table:**

	*U*^11^	*U*^22^	*U*^33^	*U*^12^	*U*^13^	*U*^23^
Cu	0.0271 (3)	0.0284 (3)	0.0217 (3)	0.0038 (2)	0.0086 (2)	0.0027 (2)
O1	0.0293 (18)	0.049 (2)	0.0288 (16)	−0.0038 (15)	0.0052 (14)	−0.0014 (15)
O31	0.0236 (17)	0.0296 (16)	0.0273 (15)	0.0019 (13)	0.0050 (13)	−0.0020 (13)
O32	0.0310 (19)	0.067 (2)	0.0319 (17)	0.0094 (17)	0.0119 (15)	−0.0013 (16)
O33	0.0377 (19)	0.0389 (19)	0.0288 (16)	0.0048 (15)	0.0104 (14)	0.0091 (14)
O34	0.058 (2)	0.045 (2)	0.0338 (18)	0.0047 (18)	0.0123 (17)	0.0152 (16)
O35	0.039 (2)	0.044 (2)	0.0291 (16)	−0.0013 (16)	0.0119 (14)	−0.0023 (14)
N11	0.031 (2)	0.033 (2)	0.0234 (18)	0.0047 (17)	0.0108 (16)	0.0030 (15)
N12	0.039 (2)	0.038 (2)	0.0264 (19)	0.0089 (18)	0.0127 (17)	0.0060 (17)
N21	0.033 (2)	0.034 (2)	0.0227 (18)	0.0052 (17)	0.0113 (16)	0.0016 (16)
N22	0.038 (2)	0.028 (2)	0.0274 (19)	0.0005 (17)	0.0130 (17)	−0.0018 (15)
C11	0.043 (3)	0.034 (3)	0.041 (3)	0.008 (2)	0.019 (2)	−0.002 (2)
C12	0.039 (3)	0.037 (3)	0.049 (3)	0.012 (2)	0.019 (2)	0.006 (2)
C13	0.030 (3)	0.036 (3)	0.035 (2)	0.007 (2)	0.013 (2)	0.007 (2)
C14	0.083 (5)	0.059 (4)	0.047 (3)	0.018 (3)	0.026 (3)	−0.009 (3)
C15	0.051 (3)	0.057 (3)	0.038 (3)	0.023 (3)	0.008 (2)	0.012 (2)
C21	0.037 (3)	0.033 (3)	0.033 (2)	0.006 (2)	0.015 (2)	0.009 (2)
C22	0.048 (3)	0.028 (3)	0.040 (3)	−0.004 (2)	0.018 (2)	0.003 (2)
C23	0.027 (2)	0.029 (2)	0.035 (2)	0.0009 (19)	0.0103 (19)	−0.0027 (19)
C24	0.081 (5)	0.057 (4)	0.038 (3)	−0.002 (3)	0.026 (3)	0.011 (3)
C25	0.043 (3)	0.042 (3)	0.038 (3)	−0.005 (2)	0.012 (2)	−0.009 (2)
C31	0.028 (3)	0.037 (3)	0.023 (2)	0.005 (2)	0.0072 (19)	0.0093 (19)
C32	0.028 (3)	0.055 (3)	0.029 (2)	−0.003 (2)	0.009 (2)	−0.003 (2)
C33	0.041 (3)	0.058 (3)	0.031 (2)	0.014 (3)	0.009 (2)	0.012 (2)
C34	0.044 (3)	0.036 (3)	0.020 (2)	0.006 (2)	0.011 (2)	−0.002 (2)
O1W	0.111 (5)	0.152 (6)	0.182 (7)	−0.049 (5)	0.062 (5)	−0.026 (5)
O2W	0.176 (8)	0.094 (5)	0.180 (7)	−0.003 (5)	0.022 (6)	0.031 (5)

### Geometric parameters (Å, °)

**Table:**

Cu—O33	1.960 (3)	C14—H14B	0.9600
Cu—N21	1.995 (4)	C14—H14C	0.9600
Cu—N11	2.015 (3)	C15—H15A	0.9600
Cu—O31	2.020 (3)	C15—H15B	0.9600
Cu—O1	2.228 (3)	C15—H15C	0.9600
O1—H1A	0.8500	C21—C22	1.360 (6)
O1—H1B	0.8500	C21—C24	1.498 (6)
O31—C31	1.263 (5)	C22—C23	1.381 (6)
O32—C31	1.246 (5)	C22—H22	0.9300
O33—C34	1.266 (5)	C23—C25	1.495 (6)
O34—C34	1.245 (5)	C24—H24A	0.9600
O35—C32	1.421 (5)	C24—H24B	0.9600
O35—C33	1.424 (6)	C24—H24C	0.9600
N11—C13	1.332 (5)	C25—H25A	0.9600
N11—N12	1.364 (5)	C25—H25B	0.9600
N12—C11	1.329 (6)	C25—H25C	0.9600
N12—H12A	0.7732	C31—C32	1.519 (6)
N21—C23	1.334 (6)	C32—H32A	0.9700
N21—N22	1.366 (5)	C32—H32B	0.9700
N22—C21	1.342 (6)	C33—C34	1.512 (7)
N22—H22A	0.7550	C33—H33A	0.9700
C11—C12	1.367 (7)	C33—H33B	0.9700
C11—C14	1.503 (6)	O1W—H1WA	0.8499
C12—C13	1.395 (6)	O1W—H1WB	0.8500
C12—H12	0.9300	O2W—H2WA	0.8499
C13—C15	1.491 (6)	O2W—H2WB	0.8748
C14—H14A	0.9600		
			
O33—Cu—N21	168.02 (14)	H15A—C15—H15B	109.5
O33—Cu—N11	88.43 (13)	C13—C15—H15C	109.5
N21—Cu—N11	91.13 (14)	H15A—C15—H15C	109.5
O33—Cu—O31	94.10 (12)	H15B—C15—H15C	109.5
N21—Cu—O31	86.76 (13)	N22—C21—C22	106.1 (4)
N11—Cu—O31	176.92 (12)	N22—C21—C24	120.3 (4)
O33—Cu—O1	87.35 (12)	C22—C21—C24	133.6 (5)
N21—Cu—O1	104.62 (13)	C21—C22—C23	107.5 (4)
N11—Cu—O1	94.36 (13)	C21—C22—H22	126.2
O31—Cu—O1	84.00 (12)	C23—C22—H22	126.2
Cu—O1—H1A	130.6	N21—C23—C22	109.6 (4)
Cu—O1—H1B	120.0	N21—C23—C25	121.4 (4)
H1A—O1—H1B	107.7	C22—C23—C25	129.0 (4)
C31—O31—Cu	122.4 (3)	C21—C24—H24A	109.5
C34—O33—Cu	125.6 (3)	C21—C24—H24B	109.5
C32—O35—C33	113.1 (4)	H24A—C24—H24B	109.5
C13—N11—N12	105.3 (3)	C21—C24—H24C	109.5
C13—N11—Cu	132.4 (3)	H24A—C24—H24C	109.5
N12—N11—Cu	120.7 (3)	H24B—C24—H24C	109.5
C11—N12—N11	111.5 (4)	C23—C25—H25A	109.5
C11—N12—H12A	124.7	C23—C25—H25B	109.5
N11—N12—H12A	122.8	H25A—C25—H25B	109.5
C23—N21—N22	105.4 (3)	C23—C25—H25C	109.5
C23—N21—Cu	131.4 (3)	H25A—C25—H25C	109.5
N22—N21—Cu	123.0 (3)	H25B—C25—H25C	109.5
C21—N22—N21	111.4 (4)	O32—C31—O31	123.9 (4)
C21—N22—H22A	131.2	O32—C31—C32	117.4 (4)
N21—N22—H22A	117.3	O31—C31—C32	118.8 (4)
N12—C11—C12	107.3 (4)	O35—C32—C31	113.2 (4)
N12—C11—C14	121.6 (4)	O35—C32—H32A	108.9
C12—C11—C14	131.1 (5)	C31—C32—H32A	108.9
C11—C12—C13	105.8 (4)	O35—C32—H32B	108.9
C11—C12—H12	127.1	C31—C32—H32B	108.9
C13—C12—H12	127.1	H32A—C32—H32B	107.7
N11—C13—C12	110.1 (4)	O35—C33—C34	114.0 (4)
N11—C13—C15	122.3 (4)	O35—C33—H33A	108.8
C12—C13—C15	127.6 (4)	C34—C33—H33A	108.8
C11—C14—H14A	109.5	O35—C33—H33B	108.8
C11—C14—H14B	109.5	C34—C33—H33B	108.8
H14A—C14—H14B	109.5	H33A—C33—H33B	107.7
C11—C14—H14C	109.5	O34—C34—O33	123.1 (5)
H14A—C14—H14C	109.5	O34—C34—C33	115.8 (4)
H14B—C14—H14C	109.5	O33—C34—C33	121.1 (4)
C13—C15—H15A	109.5	H1WA—O1W—H1WB	107.7
C13—C15—H15B	109.5	H2WA—O2W—H2WB	108.2

### Hydrogen-bond geometry (Å, °)

**Table:**

*D*—H···*A*	*D*—H	H···*A*	*D*···*A*	*D*—H···*A*
O1—H1A···O32^i^	0.85	2.21	2.798 (5)	126
O1—H1B···O32^ii^	0.85	1.97	2.764 (5)	156
O1W—H1WA···O34	0.85	1.93	2.707 (8)	151
O1W—H1WB···O35^iii^	0.85	2.45	3.097 (8)	133
O2W—H2WA···O32^ii^	0.85	2.23	3.024 (8)	156
O2W—H2WB···O1W^iv^	0.88	1.87	2.741 (10)	171
N12—H12A···O34^iii^	0.77	2.03	2.773 (5)	163
N22—H22A···O31^ii^	0.75	2.20	2.904 (5)	155

Symmetry codes: (i) *x*+1, *y*, *z*; (ii) −*x*, −*y*+1, −*z*+1; (iii) −*x*, −*y*+1, −*z*; (iv) −*x*+1, −*y*+1, −*z*+1.
